# A Twelve-Year Experience in Ambulatory Surgery within Urology

**DOI:** 10.5402/2012/383642

**Published:** 2012-02-16

**Authors:** Pedro Navalón, Yoni Pallás, Victor Navalón, Felipe Ordoño, Elisa Monllor

**Affiliations:** Urology Department, University Hospital Casa de la Salud, Catholic University, 46023 Valencia, Spain

## Abstract

*Purpose*. The aim of this study is to show you the results we obtained through the integration of the Urology Department into the Ambulatory Surgery Unit for the very first twelve years. *Scope*. We will explain both the criteria we followed for patients to join in and the surgical and anesthetic procedures we used with those 1544 patients who were ambulatory subjected to urological diseases. After those patients were treated, they reached up to 95% of reasonable results. *Conclusions*. Most of urological patients liable to have surgical treatment are bound to be included in an ambulatory surgery program, which implies neither a worse healthcare service standard nor a worse satisfaction in patients.

## 1. Introduction

Spanish healthcare service guide for surgery and organization describes ambulatory surgery (CMA) as “*Surgical procedures which are constrained to be done under general, local, regional or sedative anaesthesia or that requires [sic] few intensive postoperative and short-term cares, hence there's no need of hospital admission and patients can be discharged after some few hours of surgery*” [1]. It is an optimal model of multidisciplinary surgical attendance that allows you to treat certain patients in a very safe and effective way, with no need to get a traditional admission hospital bed [2]. This implies, therefore, that the patient can spend both the previous night and the night after surgery at home.

Although ambulatory surgery has been called in different ways, such as surgery without admission, advanced discharge surgery, day surgery and so on, it is best known in our country as “major ambulatory surgery” (in Spanish CMA). This is in order to make a difference from “minor ambulatory surgery”, which includes procedures of very low complexity and that almost never results in hospital admission, and therefore, it normally employs local anaesthesia (office based surgery).

The need to simplify and sort out the ambulatory surgery procedures has led us to develop specific units known as UCMAS's, which stands for “*the organization of sanitary professionals that supplies you with multidisciplinary attendance to processes by means of CMA, and that fulfils functional, structural and organizational requirements so as to guarantee the appropriate quality and efficient conditions to run this activity*” [3, 4].

On the other hand, the increase of the standard of living, the so-called welfare state, and the longevity of population, besides a greater offer in sanitary assistance, all this has led to a progressive increase of surgery demand, which has found out the limitation of the sanitary resources. The CMA was born in order to optimize sanitary resources, among other reasons. Therefore, not only does it make surgery be faster and optimize the increasing costs but it also improves assistance quality [5, 6].

Urology is a specialty with a large projection in the field of the CMA. On the one hand, there is a great amount of urological pathology with average complexity likely to have surgery with no need of hospital admission, and on the other, it is a specialty that in the last years it has shown a spectacular improvement in its technological resources (endoscope, shock waves, thermotherapy, laser etc). All this is leading to an evolution towards less and less invasive but simpler and safer surgical procedures and therefore with less need of hospital admission [7].

Although the urological treatments included in mostly published guides (Ministerio de Sanidad y Consumo of Spain, Royal College of surgeons of England…) are limited to vasectomy, transurethral cystoscopy, urethral dilatation, extracorporeal lithotripsy, circumcision, scrotal pathology treatment, orchidectomy, surgery of hydrocele, orchidopexy, and meatoplasty, this list might appear to be short compared to the wide range described in the literature and in our own experience itself.

In order to unify criteria, “*Conselleria de Sanidad de la Comunidad Valenciana*” [8] which is our regional healthcare service, published recently a list of urologic surgery procedures which were subjected to be done within ambulatory surgery. It included more than thirty different types of surgical procedures, both inguinoscrotal pathology, surgery of penis and urogynecological, endoscopic, and percutaneous surgeries. Those urologic surgical procedures subjected to be done in ambulatory surgery are detailed in Table 1.

The aim of this publication is to show you those results obtained after our first twelve years from running the service of urology integrated within the UCMA of our hospital.

## 2. Contents and Methods

Between January of 2000 and July of 2011 (12 years) in the UCMA of our hospital, we subjected, in strictly day surgery, 1544 patients affected by urological pathology to surgery: inguinoscrotal, urethrovesical, penis, and stress incontinence.

Selection criteria were set by considering the following features: surgical procedure to be made, the overall patient physical conditions (anaesthesia and surgical risk), patient's features, and his social environment.

On defining the surgical procedures to be included, we took into account those aspects detailed in Table 2. As far as the overall physical condition of the patient was concerned, we turned to those criteria of surgical anaesthetic risk set by the American Association of anesthesiologists (ASA). We included patients qualified as ASA I, ASAII, and ASA III good compensated at the moment of surgery. Regarding patient's profile, the main condition we focused on was for him to agree with such surgery program without hospital admission. In addition, such patient should be able to cooperate and fully understand medical instructions, and what is more, the patient should also assume any postoperative upset. Consequently, we considered it very important to provide the patient with a great amount of information regarding the whole process. The last characteristic we valued, in order to proceed with patient selection, was its standard of living or social environment as follows: he should have a house in proper conditions (i.e., with elevator, telephone…); a responsible adult would be needed to take care for the patient for the first 24–48 hours of aftercare (postoperative) and an available means of transport. The distance from his home to the hospital should be such that it took no longer than 30–45 minutes by car.

None of the treated patients needed preoperative hospital admission. They just came right from their homes into the UCMA at the scheduled time and day unfed, dressed in comfortable clothes, and have had a thoroughly shower.

The types of anaesthesia used were as follows: local anaesthesia + sedation or general one with larynx mask.

After surgery, patients were transferred to the Postanaesthesic Recovery Unit I (URPA I), next to the operating theatre, where they remained for between 1 or 2 hours monitored by nurse staff. They were transferred later to the Postanaesthesic Recovery Unit II (URPA II), where they remained beside a relative, until those discharging criteria detailed in Table 3 were fulfilled.

On discharging the patient from hospital, we provided both the patient and his relative with a discharge summary related to that specific surgery, where all cares that should be taken at home were detailed such as wound care, recommended hygiene, recommended diet during the first hours and the following days, recommended analgesia and antibiotherapy procedure in case it would be needed, date for next checkup, and a 24-hour assistance telephone.

Medical and nurse staff phoned the patients after surgery just the same afternoon as the surgery took place and the following next morning too. This was done in order to cater for his stage and, consequently, avoid possible complications to occur and fill the patient in confidence. This will lower the feeling of lack of continuous assistance that this ambulatory procedure might lead to.

We made the first checkup generally the second day after surgery. The successive ones were scheduled depending on the kind of pathology and specific surgery.

We made an anonymous survey on the patient satisfaction degree according to the corresponding treatment received by each one.

## 3. Results

Out of the 1544 patients operated on, 1338 were male (91%) and 146 (9%) women, with an average age of 51.2 years (range from 3 to 88).

We did antibiotic prophylaxis in 1404 (91%), by using monodosage guidelines previous to surgical procedure, by using cephalosporin in 968 (69%) patients, quinolones in 244 (16%), and amoxicillin-clavulanic in 212 (15%).

The type of anaesthesia used was local anaesthesia + sedation in 1278 (83%) patients, general anaesthesia with larynx mask in 254 (16%), and rachideal anaesthesia in 12 (1%).

The surgical procedures carried out are detailed in Table 4.

From the amount of the whole operated patients, only 16 of them (1%) required hospital admission due to immediate complications during their staying in the UCMA, which we proceed to detail a 5-year-old child that was operated on with cryptorchidism by suffering from a bronchospasm, a 17-year-old patient who was affected of varicocele because of vomiting and pain in the inguinal area, and other 12 elderly patients, because of a large bleed episode after transurethral resection of a bladder tumour. All of them were discharged in the first 24 hours after the intervention.

Thirty patients (2%) were assisted at emergency department during the immediate postoperative (first 24 hours), twelve of them due to intense pain in the surgical wound, which was resolved by increasing oral analgesia, and in 4 of them anxiolytic treatment was also added. The other 18 patients (12 phimosis in children and 6 hydrocele) showed themselves due to wound bleeding. There was only one of them that got an important scrotal haematoma. This patient needed hospital admission and surgical exploration.

During the first postoperative week, 42 patients (3%) showed some sort of complications: 18 epididymitis after probing or following hydrocelectomy, 16 surgical wound infections, and 8 urine retentions after the bladder catheter was removed. All of them were solved out with conservative treatment at their homes.

Late complications also occurred. Those were the ones related to the own and inherent type of surgery carried out in each patient regardless getting through ambulatory surgery or not. That's why we do not mention them since they are off the scope for the present study.

An amount of 1498 patients (97%) answered the survey that valued their degree of satisfaction related to the received treatment and whose results are detailed in Table 5.

## 4. Discussion

It is a fact, and mainly in the last years, that CMA heads an unstoppable lead; so, in the United States, 60% out of the whole surgery acts are carried out through ambulatory surgery. This country is the one where this organizing system for surgical assistance has been developed more thoroughly [7, 9].

Urology is an excellent specialty to use this sort of assistance method due to its increasing and spectacular improvement of technological means and the existence of a large number of processes capable of being carried out with no need of hospital admission, as far as our experience demonstrates.

We agreed with other authors [9, 10] that CMA sets up an assistance system where all members get profit from. There is a sanitary cost reduction for the hospital, which varies from 25% up to 75% [11–13]. This is due to the fact that, even though the cost of the surgery is similar to that of hospital admission, the savings are obtained mainly in hostelry and nursing staff. In addition, the number of free beds increases since few patients that get surgery must end up in hospital admission.

The patient also gets some benefits, as he comes home a few hours after being operated on and hence life is disrupted for less time. This decreases both patient anxiety and the out-break of complications related to hospital accommodation such as nosocomial infection and the secondary infection due to being bedridden [9]. Patient satisfaction is also unusually high, as our survey stands out with a 96% of the results considered to be excellent (73%) and good (23%). These results are similar to the ones obtained in the set of patients who did not receive ambulatory care but rather were in hospital for postoperative care.

When ambulatory surgery was born, urologists considered it to be as a “minor surgery.” This unjustified belief was carried on for years. However urologist had already been using some methods of ambulatory surgey in order to decrease risks. As a result, those methods had helped the patient to come home some few hours after of the surgery. Such methods mentioned before could be as follows: applying a lidocaine cream and prilocaine 1 hour before the surgery to anesthetize skin, using xilocaine gel to 2%, 20–30 minutes before for endoscopic surgery, infiltrating the base of a bladder tumour with endoscope needle for his resection with local anaesthesia, the infiltration of the spermatic cord with mepivacaine or lidocaine, doing small incisions, getting a thoroughly haemostasis, or applying prophylaxis for the postoperative pain, and so forth. This has turned out to be very rewarding and satisfying.

The obtained results, similar to those of the hospital admitted patients, showed a low incidence of complications, all of them minors, with resumption of motivation and professional satisfaction. All this led us to our present *“working philosophy”* which pushes us to widen our experience and career in ambulatory procedures, and to include steadily higher-complexity pathologies [14–19].

## Figures and Tables

**Table 1:** List of urologic procedures subjected to be carried out through CMA.

*INGUINO-ESCROTAL PATHOLOGY*	
Orchidopexy	Hydrocele surgical treatment
Orchidectomy	Varicocele surgical treatment
Epididymectomy	Cordon cyst surgical treatment
Placing of testicle prothesis	Treatment of peritonea-vaginal process
Testicle Biopsy	

*PATHOLOGY OF PENIS*	
Meatoplasty	Partially Penectomy*
Paediatric circumcision	Venous leak treatment
Nesbit procedure*	

*PERCUTANEOUS PROCEDURES*	
Percutaneous nephrostomy	Suprapubic cystostomy
Simple renal cyst punction	Perineal prostatic biopsy
Percutaneous renal biopsy	Extracorporeal shock wave lithotripsy (ESWL)

*ENDOSCOPIC PROCEDURES*	
Endoscopic Urethrotomy*	Placing of urethral stents
Transurethral incision of the prostate TUIP*	Placing and removal of urethral stents
TUR of bladder tumour*	Uretheroscopy
Bladder biopsy	Endoscopic urethral Lithotripsy*

*UROGYNECOLOGY*	
Urethral caruncle surgery	Specific procedures for stress UI

*Selected cases.

**Table 2:** Criteria to include surgery procedures.

No need for complex setup	
Least risk for bleeding episodes	
Surgery time less than 90 minutes	
Postoperative pain controlled by oral analgesia	
No need of drains with high outflow	
Fast oral tolerance	
Liable for fast mobility	

**Table 3:** Discharge criteria.

Conscious and oriented	Control of pain
Steady vital signs during the last hour	Autonomous mobility without dizzy sensation

Liquid tolerance	Lack of bleeding

Spontaneous diuresis	Lack of vomiting and pain

**Table 4:** List of the proceeded surgery.

*Inguinoescrotal surgery*	No. of cases	%
Hydrocelectomy	538	35%
Cord Cystectomy	214	14%
Varicocelectomy	170	11%
Orchidopexy	49	3%
Treatment peritoneal vaginal process	44	3%
Orchidectomy	24	2%

*Uretrovesical surgery*		
TUR of small bladder tumours	120	8%
Endoscopic urethrotomy	75	5%
Carunclectomy	24	2%
TUIP-PTUR	36	2%

*Surgery of penis*		
Phymosis in children	80	5%
Nesbit procedure	64	4%
Balanic hypospadias	30	2%

*Stress IU female surgery*		
TVT	76	5%

**Table 5:** Satisfaction with the received treatment.

Score	No. of cases	%
Excellent	1090	73%
Good	342	23%
Fair	52	3%
Poor	14	1%

## References

[1] Ministerio de Sanidad y Consumo-Dirección General de Aseguramiento y Planificación Sanitaria (1993). *Cirugía Mayor Ambulatoria: Guía de Organización y Funcionamiento*.

[2] Martín Morales J (1996). Cirugía mayor ambulatoria: una transformación necesaria. *Cirugía Mayor Ambulatoria*.

[3] Giner M, Porrero JL (1999). Acreditación en unidades de Cirugía Mayor ambulatoria. *Cirugía Mayor Ambulatoria. Madrid, Manual Práctico*.

[4] Marin J, Esteban S (1988). Ambulatory Surgery in Spain. *Ambulatory Surgery*.

[5] De Lathouwer C, Poullier JP (2000). How much ambulatory surgery in the World in 1996-1997 and trends?. *Ambulatory Surgery*.

[6] Jarrett P, De Lathouwer C, Ogg TW (2000). The time has come to promote true day surgery. *Ambulatory Surgery*.

[7] Rodríguez JM, Rodríguez R, Blanco G, Porrero JL (2002). Cirugía mayor ambulatoria en urología. *Cirugía Mayor Ambulatoria*.

[8] Generalitat Valenciana (2002). *Guía de Actuación en Cirugía Mayor Ambulatoria*.

[9] Davis JE (1987). Futuro de la Cirugía mayor ambulatoria. *Clin Quir Norteam*.

[10] Llopis Guixot B, Navarro Antón JA, Mola Arizo MJ (2003). Cirugía mayor ambulatoria en urología: 5 años de experiencia. *Actas Urologicas Espanolas*.

[11] Caldemone A, Rabinowitz R (1990). Outpatient orchiopexy. *Journal of Urology*.

[12] Sadler GP, Richards H, Watkins G, Foster ME, Atwell JD (1992). Day-case paediatric surgery: the only choice. *Annals of the Royal College of Surgeons of England*.

[13] Vargas Blasco C, Rius Espina G (1993). Cirugía urológica sin ingreso. *Actas Urologicas Espanolas*.

[14] Navalón P, Zaragoza C, Pallas Y Urología: una especialidad con gran proyección en cirugía mayor ambulatoria. http://www.siicsalud.com/dato/dat042/05222015.htm.

[15] Navalón P, Zaragoza C, Pallas Y Buena costoefectividad de la cirugía mayor ambulatoria en urologia. http://www.siicsalud.com/dato/dat050/06905001.htm.

[16] Navalón P, Pallas Y, Ordoño F La resección transuretral de próstata bajo anestesia local y sedación es segura y bien tolerada. http://www.siicsalud.com/dato/experto.php/101173.

[17] Navalón Verdejo P, Zaragozá C, Ordoño F (2005). Tratamiento del hidrocele en cirugía mayor ambulatoria. *Archivos Espanoles de Urologia*.

[18] Navalón P, Zaragozá C, Sánchez F (2005). Corrección del pene curvo en cirugía mayor ambulatoria. *Actas Urologicas Espanolas*.

[19] Navalón P, Zaragozá C, Ordoño F (2005). Tratamiento quirúrgico ambulatorio de la incontinencia urinaria de esfuerzo femenina. *Archivos Espanoles de Urologia*.

